# Novel Application of Metal–Organic Frameworks as Efficient Sorbents for Solid-Phase Extraction of Chemical Warfare Agents and Related Compounds in Water Samples

**DOI:** 10.3390/molecules29143259

**Published:** 2024-07-10

**Authors:** Jakub Woźniak, Stanisław Popiel, Jakub Nawała, Barbara Szczęśniak, Jerzy Choma, Dariusz Zasada

**Affiliations:** Institute of Chemistry, Faculty of Advanced Technologies and Chemistry, Military University of Technology, Kaliskiego Str. 2, 00-908 Warsaw, Poland; jakub.wozniak01@wat.edu.pl (J.W.); jakub.nawala@wat.edu.pl (J.N.); barbara.szczesniak@wat.edu.pl (B.S.); jerzy.choma@wat.edu.pl (J.C.); dariusz.zasada@wat.edu.pl (D.Z.)

**Keywords:** metal–organic framework, solid-phase extraction, chemical warfare agents, gas chromatography–tandem mass spectrometry, water samples

## Abstract

In this work, we test metal–organic frameworks (MOFs) as sorbents in the solid-phase extraction (SPE) technique to determine chemical warfare agents (CWAs) and their related compounds in water samples. During this study, we used 13 target compounds to test the selectivity of MOFs thoroughly. Three MOFs were used: MIL-100(*Fe*), ZIF-8(*Zn*), and UiO-66(*Zr*). The obtained materials were characterized using FT-IR/ATR, SEM, and XRD. CWA’s and related compounds were analyzed using gas chromatography coupled with tandem mass spectrometry (GC-MS/MS). The effect of the type of elution solvent and the amount of sorbent (MOFs) in the column on the efficiency of the conducted extraction were verified. The LOD ranged from 0.04 to 7.54 ng mL^−1^, and the linearity range for the analytes tested extended from 0.11/22.62 (depending on the compound) to 1000 ng mL^−1^. It was found that MOFs showed the most excellent selectivity to compounds having aromatic rings in their structure or a “spread” spatial structure. The best recoveries were obtained for DPAA, CAP, and malathion. Environmental water samples collected from the Baltic Sea were analyzed using an optimized procedure to verify the developed method’s usefulness.

## 1. Introduction

The interest in conducting chemical analyses of chemical warfare agents (CWAs) does not lose its importance. In 1997, the Chemical Weapons Convention (CWC) was implemented [1,2,3]. The convention prohibits research into the development of toxic warfare agents, bans the use of chemical weapons using such compounds, and mandates the successive destruction of chemical weapons stockpiles by member countries [2,4]. There is also the issue of the thousands of tons of chemical munitions dumped in the seas and oceans after World War II under provisions agreed upon at the 1945 Potsdam Conference [5]. The chemical weapons found in the Baltic Sea still pose a threat to both the local ecosystem and people [6,7]. There also remains the problem of terrorist attacks using the CWAs [8,9] and munitions abandoned on military training grounds. For the reasons mentioned above, a crucial issue is the development of methods for analyzing complex samples from diverse environments.

The determination of CWAs and their degradation products in environmental samples are challenging, for example, due to the usually low concentrations of the compounds determined, the presence of interferents, the different nature of analytes detected, or often their instability in aqueous environments [10,11,12,13]. The development of new and the improvement of existing techniques for preparing and analyzing samples containing CWAs and their degradation products is still underway [14]. These techniques require constant refinement and development. One of the most popular techniques for preparing liquid samples for chromatographic analysis is solid-phase extraction (SPE). This technique is based on the sorption of analytes present in liquid samples on a solid sorbent, followed by their elution with a suitable organic solvent, allowing for multiple concentrations of the analytes under study, removal of interferents, and alteration of the sample matrix. Several commercial sorbents are available on the market for use in this technique. However, work is still underway to develop a new sorbent or improve an existing sorbent that will be more efficient or selective toward selectively choosing the studied analytes [15,16,17].

Metal–organic frameworks (MOFs) are a group of functional materials that find application in more and more fields of science and technology. The first reports of MOFs appeared in the early 1990s [18,19]. MOFs are built from two elements—a metal ion or cluster and an organic linker. A coordination bond connects these two elements; they form an ordered, porous spatial structure with a specific surface area reaching up to 7000 m^2^ g^−1^ (several times that of even the best-activated carbon). MOFs have found their applications mainly in gas storage and separation, as catalysts in chemical reactions and drug carriers, and are also used in chemical sensors [20,21,22]. An emerging but promising use of MOFs is as sorbents in various sample preparation techniques for analysis [23,24]. In recent years, there have been an increasing number of scientific publications describing the use of MOFs as sorbents, mainly, but not only in the SPE technique, for the determination of common organic pollutants present in the environment.

As far as we know, the sorption properties of MOFs toward CWA have never yet been investigated in a similar way to the one we presented. No one has previously tested the feasibility of using MOFs as sorbents to determine CWA. This very fact was our primary motivation for this article. Analyzing the literature on MOFs and CWA, we encountered mostly articles on the neutralization of CWA using MOFs or those in which the authors use other properties of MOFs but do not use these materials as typical sorbents in the SPE technique [25]. This topic has been of particular interest to us because these materials can be hypothetically synthesized and modified in a wide range, potentially allowing for the creation of MOFs that will have specific sorption properties (both efficiency and selectivity) against selected CWA or any other compounds. We wanted to develop a quick, easy-to-use, inexpensive method for determining these compounds. We are aware that most of the described methods of MOF synthesis are based on using a Teflon-lined autoclave, and the presented synthesis methods are time-consuming; some may take up to several days [26]. MOFs synthesis can also be carried out using basic laboratory glassware available in every laboratory and performed relatively rapidly. This approach may contribute to the practicality of the developed method. However, the primary purpose of this article was not to create a new material but to practically test the applicability of selected MOFs synthesized in a facile way for the determination of CWAs and their related compounds and to develop a more straightforward, faster, and inexpensive analytical method.

In theory, syntheses of hundreds of different MOFs could be planned, which would allow for matching the material properties to a specific analyte or group of analytes. However, in practice, there is a problem with the applicability of those hypothetically predicted syntheses and the durability of MOFs in aqueous environments. Not every planned reaction is possible to conduct, and some MOFs can degrade/hydrolyze in aqueous environments. Luckily, some research is being undertaken to develop MOFs that show increased durability in the aquatic environment [20,27]. In recently published work on using MOFs as sorption materials, one can observe a clear interest in MOFs referred to as MIL—Materials from Institute Lavoisier, ZIF—Zeolitic Imidazolate Framework, and UiO—University of Oslo. Specific examples include MIL-100(*Fe*) [28], MIL-100(*Cr*) [29], MIL-53(*Al*) [30], ZIF-8(*Zn*) [31], UiO-66(*Zr*) [32], and UiO-67(*Zr*) [33]. According to the authors of these works, the mentioned materials can be successfully used as sorbents, among others, in the SPE technique to prepare various samples to determine several compounds, mostly common organic pollutants found in the environment. According to the results presented in the literature, MOFs referred to as “MIL”, “ZIF”, and “UiO” also show good resistance to water.

For this study, three MOFs were selected: MIL-100(*Fe*), ZIF-8(*Zn*), and UiO-66(*Zr*). Synthesized materials were used in the SPE technique to analyze water samples spiked with selected CWA, their simulants, and degradation products: dibutyl sulfide (DBS), thiodiglycol (TDG), thiodiglycol sulfoxide (TDGO), 1,4-dithiane, 1,4-thioxane, diphenylarsinic acid (DPAA), triphenylarsine oxide (TPA-O), phenylarsonic acid (PAA), chloroacetophenone (CAP), lewisite I, trimethyl phosphate (TMP), triethyl phosphate (TEP), and malathion. The analysis was conducted using gas chromatography coupled with the tandem mass spectrometry technique (GC-MS/MS). Several factors influencing the conducted extraction process were studied as part of the optimization. Selected validation parameters of the developed method were determined. The results show that the chosen MOFs can find their application as sorbents in the SPE technique in the course of determining CWAs, their simulants, and degradation products occurring in aqueous samples at very low concentration levels.

## 2. Results and Discussion

### 2.1. Characterization

**Figure 1:**
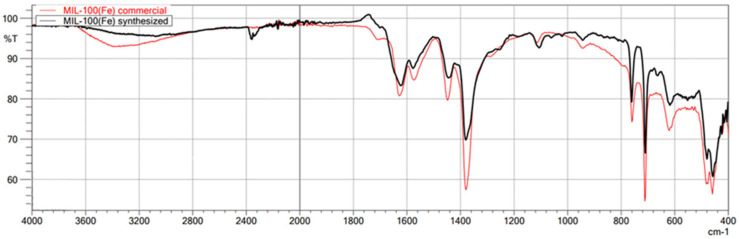
FT-IR/ATR spectrum of the obtained MIL-100(*Fe*). Comparison of spectra of synthesized and commercial MOFs.

**Figure 2:**
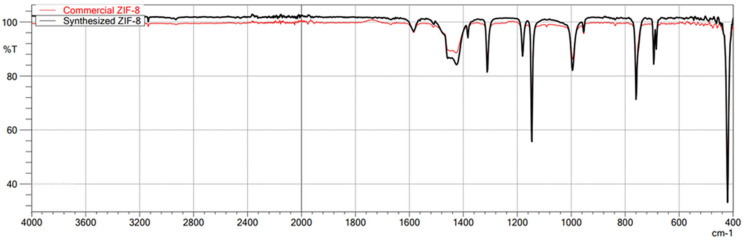
FT-IR/ATR spectrum of the obtained ZIF-8(*Zn*). Comparison of spectra of synthesized and commercial MOFs.

**Figure 3:**
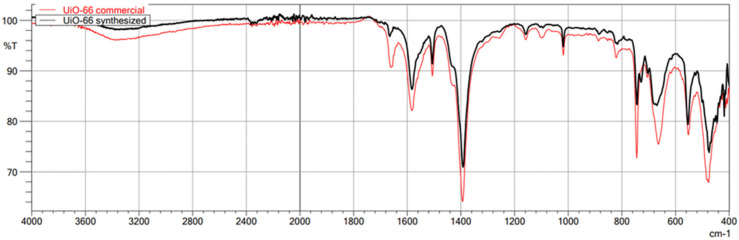
FT-IR/ATR spectrum of the obtained UiO-66(*Zr*). Comparison of spectra of synthesized and commercial MOFs.

The first technique used to confirm the structure of the obtained MOFs was FT-IR/ATR. The FT-IR technique was used here as an auxiliary for ASAP and XRD. Due to its nature, FT-IR is not the most suitable technique for quantitative research; it is undoubtedly more appropriate for qualitative research. Therefore, less attention was paid to the intensity of individual bands in the obtained spectra, and the focus was on the very fact of their presence and the wavenumber value. This study used this technique to confirm the presence or absence of the chemical bonds that make up the structure of metal–organic frameworks. This method is beneficial for comparing the spectra of the obtained MOFs with those of commercial MOFs, which was achieved in this study. The obtained spectra of the synthesized MIL-100(*Fe*) are very similar to those of the commercial MIL-100(*Fe*) (Figure 1). In the case of both materials, the identity of the absorption bands can be observed, which are characterized by the same wavenumber values in cm^−1^, differing only in the transmittance values percentage. Comparison of the synthesized MIL-100(*Fe*) spectra with the spectrum of the substrate-Trimesic acid confirms the occurrence of a chemical reaction, leading to MIL-100(Fe) formation. The data obtained indicate good agreement between the synthesized and commercial MIL-100(*Fe*). The spectra of synthesized and commercial ZIF-8(*Zn*) are almost identical (Figure 2). Comparison of the synthesized ZIF-8(*Zn*) spectra with the spectra of the substrates used during the synthesis confirms the reaction leading to the formation of ZIF-8(*Zn*). As with the other two materials, the IR spectra obtained for UiO-66(*Zr*) indicate good agreement between the commercial and synthesized MOFs (Figure 3). There is one exception: one band at 1700 cm^−1^ shows a noticeably lower intensity for the synthesized material, which may indicate an incomplete match between the obtained MOF and commercially available UiO-66(*Zr*). This may indicate that the MOF synthesis process did not go entirely according to plan. The comparison of the obtained spectra with those of the substrates used confirms the occurrence of a chemical reaction leading to the formation of UiO-66(*Zr*).

**Figure 4:**
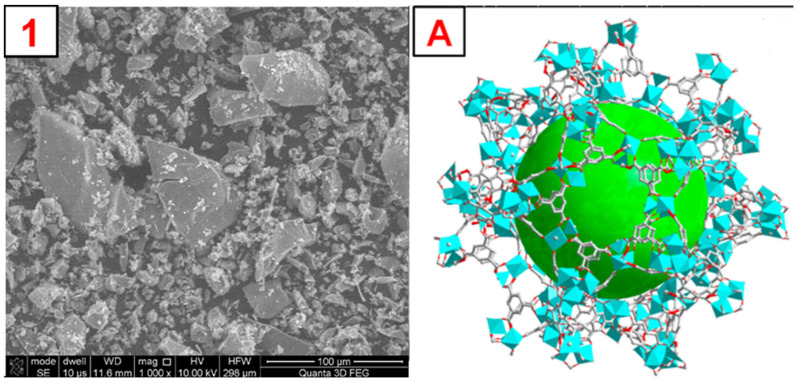
1—SEM photo of the synthesized MIL-100(*Fe*). A—a schematic representation of the MIL-100(*Fe*) structure, the green sphere inside the structure represents the space inside the pore [37].

**Figure 5:**
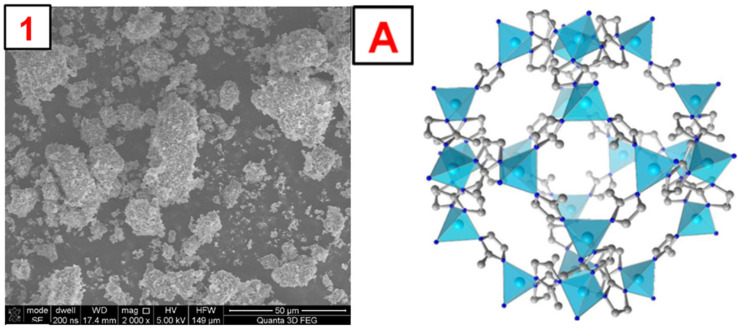
1—photo of the obtained ZIF-8(*Zn*). A—a schematic representation of the ZIF-8(*Zn*) structure [38].

**Figure 6:**
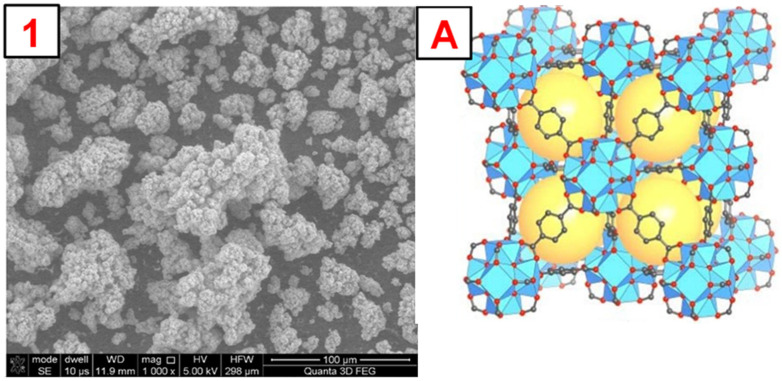
1—SEM photo of the obtained UiO-66(Zr). A—a schematic representation of the UiO-66(Zr) structure, the yellow sphere inside the structure represents the space inside the pore [20].

To illustrate the morphology of the synthesized MOFs, MIL-100(*Fe*), ZIF-8(*Zn*), and UiO-66(*Zr*), images were taken using a scanning electron microscope (SEM). The obtained photos, along with a schematic representation of the MOF structure and images obtained by other authors, are shown in Figure 4, Figure 5, Figure 6 and Figures S1–S3. The SEM MIL-100(*Fe*) images (Figure 4) show that the obtained particles are not homogeneous. There are many particles with sizes below 5–10 µm and a few with sizes up to 100 µm. On a macro-scale, larger particles differ in shape from the MOF presented in the literature [34]. However, the smaller ones show remarkable similarity. Nevertheless, it is noticeable that the forming particles of material tend to form larger crystals. SEM images of ZIF-8(*Zn*) (Figure 5) show many particles with sizes up to about 50 µm, characterized by a highly developed surface. In the microscale, the obtained particles show a remarkable similarity to the ZIF-8(*Zn*) presented in the literature [35]. Clusters of tiny grains of this MOF are visible. The photos of the UiO-66(*Zr*) (Figure 6) show the partial distribution of the obtained MOF particles into those with sizes up to about 100 µm and those with dimensions in the range of several tens of µm. On a macro scale, it is almost identical to that presented in the literature [36]; however, on the microscale, numerous small spike-like structures are visible on the surface of the synthesized material, which indicates differences in crystal structure compared to commercial UiO-66(*Zr*). This may be due to the disturbed crystallization of MOFs during its precipitation in the reaction system during synthesis.

**Figure 7:**
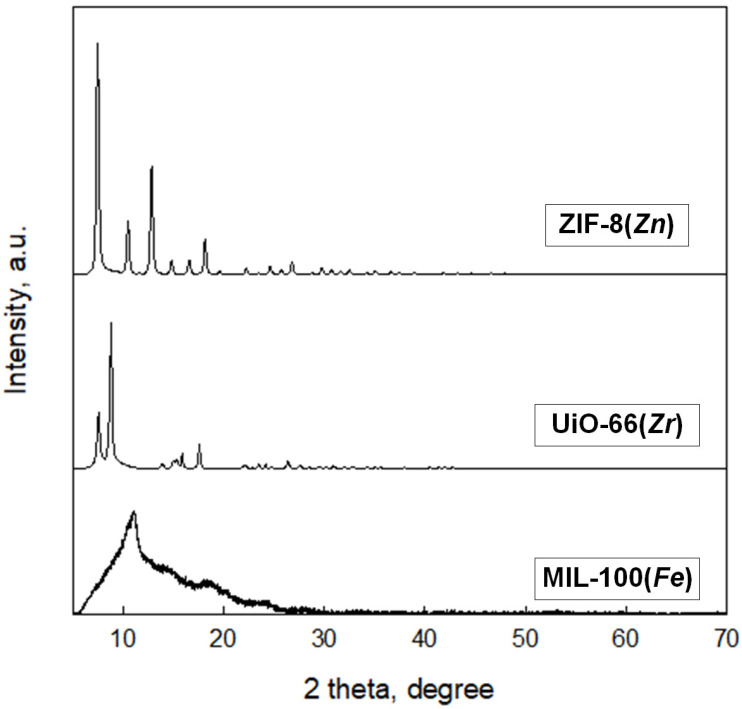
XRD spectrum obtained for the synthesized MOFs.

XRD patterns of MOFs as crystalline materials show characteristic peaks, which in the case of ZIF-8(*Zn*) and UiO-66(*Zr*) are consistent with the literature data and are presented in Figure 7 [34,35,36]. Unfortunately, the XRD pattern of the MIL-100(*Fe*) differs from the XRD patterns presented in the literature for this material. This may indicate a certain degree of amorphousness of this material compared to commercially available MOFs. Nevertheless, further data on the analysis of this material’s specific surface area and pore size are comparable to the rest of the materials obtained.

**Figure 8:**
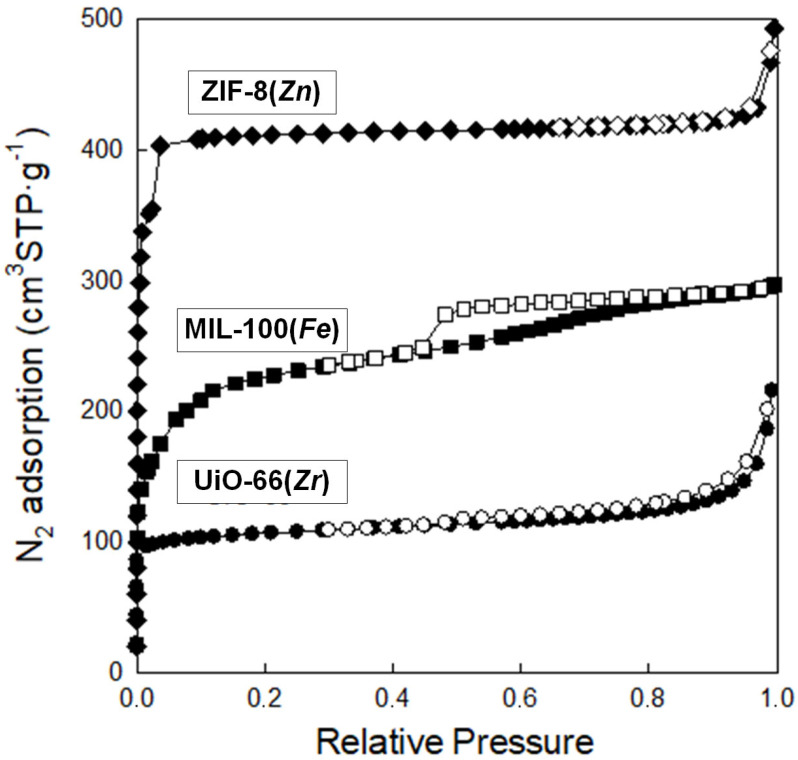
Nitrogen adsorption–desorption isotherms at −196 °C on the synthesized MOFs.

**Figure 9:**
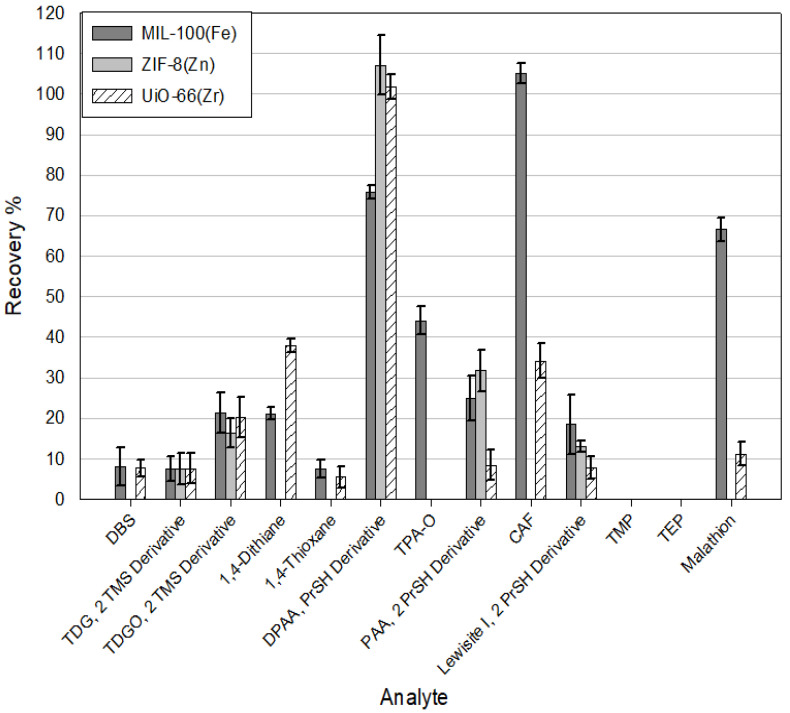
Recoveries of analytes from spiked water samples using three MOFs (MIL-100(*Fe*), ZIF-8(*Zn*), and UiO-66(*Zr*)), Elution solvent = Acetonitrile.

**Figure 10:**
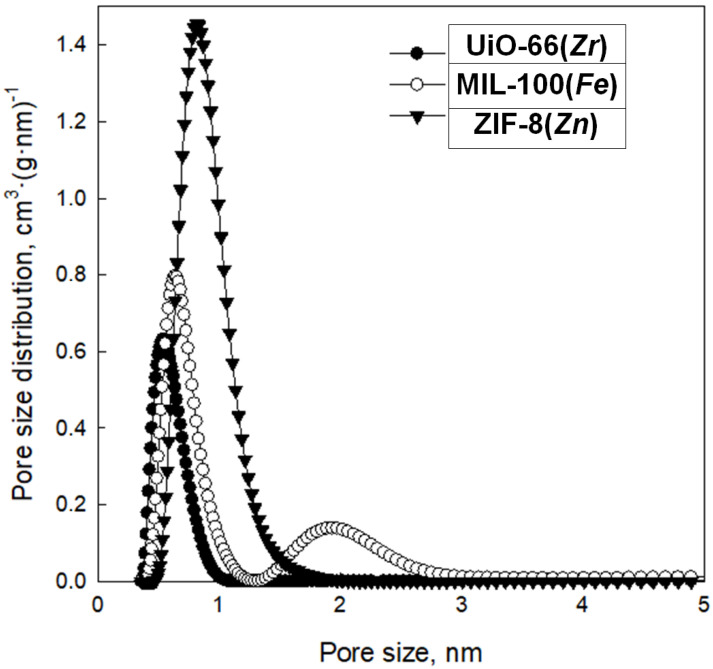
Pore size distribution functions determined for the synthesized MOFs.

Analysis of low-temperature nitrogen adsorption isotherms was used to determine structural parameters such as specific surface area (S_BET_) and pore volume (V_t_), as well as pore size distribution (PSD). Figure 8 shows nitrogen adsorption–desorption isotherms measured at −196 °C, and Figure 9 presents the corresponding PSD functions determined for the synthesized samples. These MOFs feature high S_BET_ ranging from 392 to 1479 m^2^ g^−1^; the highest value features ZIF-8(*Zn*), with a lower value of 805 m^2^ g^−1^, possesses MIL-100(*Fe*), whereas the lowest value exhibits UiO-66(*Zr*). All MOFs show large pore volumes with most micropores in their structures; V_t_ of ZIF-8(*Zn*), MIL-100(*Fe*), and UiO-66(*Zr*) are 0.76, 0.46, and 0.33 cm^3^ g^−1^, respectively. The maxima of the PSD peaks (Figure 10), and thus the average pore sizes, determined for all samples studied, are in the microporous range, up to ~2 nm (Table 1).

The data obtained show excellent agreement on both the specific surface and pore parameters for the obtained ZIF-8(*Zn*) compared with commercial material, and discrepancies for the MIL-100(*Fe*) and UiO-66(*Zr*), probably due to the poor reproducibility of their synthesis methods resulting from the necessity to maintain precise synthesis conditions for highly crystalline and porous products in the case of these two MOFs. The specific surfaces of MIL-100(*Fe*) and UiO-66(*Zr*) were found to be lower than expected, and their pore sizes also differed from those anticipated. Nevertheless, the obtained materials show a relatively high specific surface area, a certain particular crystalline structure, and small pores (on the order of 0.5–2.0 nm), which qualifies them as interesting sorption materials, e.g., in SPE techniques.

### 2.2. Effect of Desorption Conditions

The selection of the appropriate elution solvent has a crucial impact on the process of desorption of analytes from sorbents and, thus, the recovery of compounds obtained as a result of the analysis. This experiment examined the effect of four popular organic solvents (acetonitrile, ethyl acetate, dichloromethane, and hexane) on extraction efficiency. The results for the individual solvents are ranked according to their decreasing polarity. An example of experimental data is shown in Figure 10. The results show that the highest efficiency of the extraction is achieved with the use of acetonitrile. As the polarity of the solvents used decreased, the recoveries of the analytes gradually reduced as well. The best recoveries were obtained using acetonitrile as an elution solvent for DPAA, CAP, and malathion for selected MOFs. Analyzing the received data, it can be seen that the used MOFs show the most excellent affinity for DPAA, CAP, malathion, and to a lesser extent also for TDGO, 1,4-dithiane, and 1,4-thioxane. It is also worth noting that in no case were MOFs able to extract TMP and TEP. Minor recoveries were also recorded each time for DBS and TDG.

### 2.3. Effect of Amount of the MOF in SPE Columns

**Figure 11:**
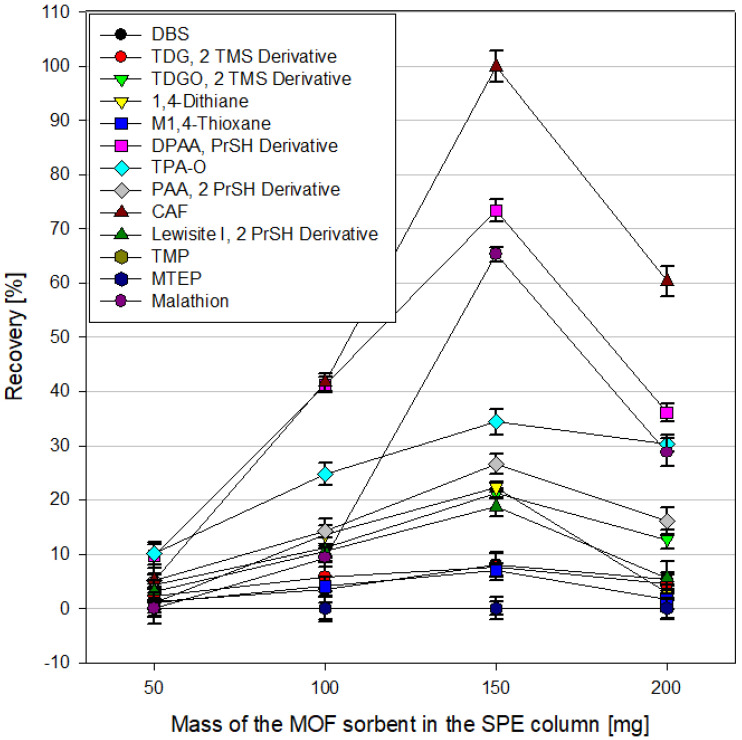
Recoveries from spiked water samples using different MIL-100(*Fe*) amounts in the SPE column.

The amount of sorbent used in the SPE column can be of great importance for the efficiency of extraction of analytes from the tested samples. On the one hand, a more significant amount of sorbent means a larger available surface on which the tested analytes present in the samples can sorb. On the other hand, it may also hinder the elution of test compounds using organic solvents. In the case of the SPE technique, the back pressure phenomenon should also be considered. The flow of the sample and organic solvents through the sorbent bed in the SPE column is forced by the reduced pressure generated by the vacuum manifold to which the columns are connected. An immense amount of sorbent usually contributes to increased back pressure, which negatively affects the flow rate of the sample and solvents through the column. In extreme cases, it completely prevents liquid from flowing through the column. This effect also depends on the grain’s size and the sorbent’s packing method. In this experiment, the dependence of analyte recoveries during SPE extraction for contaminated water samples on the amount of MOF sorbent used in the column for MIL-100(*Fe*), ZIF-8(*Zn*), and UiO-66(*Zr*) was examined for the amounts of MOF 50, 100, 150, and 200 mg.An example of these data is shown in Figure 11. The elution solvent used was acetonitrile. In the case of MIL-100(*Fe*), the highest recoveries of analytes were obtained using 150 mg of sorbent in the column; for UiO-66(*Zr*), it was 100 mg. Further increasing the amount of sorbent did not improve the obtained results while significantly increasing the back pressure. In the case of ZIF-8(*Zn*), the continuous increase in the amount of MOF used led to increased recoveries, but the study was stopped at 200 mg due to the increased back pressure and the lengthening of the time of passing the sample through the column.

### 2.4. Method Validation

**Figure 12:**
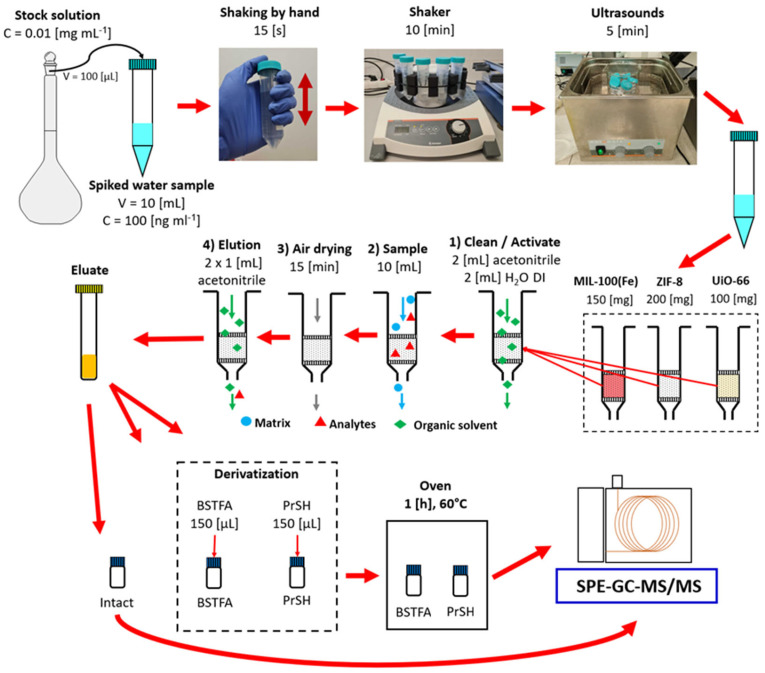
Procedure for preparation of spiked water samples and their analysis using MOF-SPE.

The optimum conditions for the SPE-GC-MS/MS method for determining selected CWAs, their simulants, and degradation products in water samples using MOFs as sorbents are summarized in Figure 12. Selected validation parameters were determined. Samples were repetitively (*n* = 5) analyzed to determine the limits of detection and quantification (LODs, LOQs), linearity range, coefficients of determination (R^2^), precision (expressed as coefficient of variation, CV, or relative standard deviation RSD, *n* = 7, intra-day). The linearity was studied over a concentration range of 0.11/22.62 (depending on the compound) to 1000 ng mL^−1^ for the 13 CWAs and the related analytes, with coefficients of determination (R^2^) ranging from 0.9900 to 0.9999. The intra-day (RSD) for seven analyses of CWA and related analytes (10 ng mL^−1^ and 50 ng mL^−1^ for TPAO) were in the range of 1.18–8.45%. The LODs for determined analytes calculated, including signal-to-noise (S/N) ratio 3:1, ranged from 0.04 to 7.54 ng mL^−1^. For the optimized method for determining selected CWAs, their simulants, and degradation products in water samples, using the SPE-GC-MS/MS technique, MIL-100(*Fe*), ZIF-8(*Zn*), and UiO-66(*Zr*) as sorbents in SPE columns in spiked water samples, the recoveries were determined. The recoveries were determined at two concentration levels for each target analyte in spiked water samples, 10 and 20 ng mL^−1^ (50 and 100 ng mL^−1^ for the TPA-O). The best recoveries were obtained for DPAA (~103%) and CAP (~100%). malathion is also worth mentioning, with recovery at ~67%. The exact results are presented in Table 2 and Table 3.

### 2.5. Analysis of an Environmental Sample—Water from the Baltic Sea

To validate the suitability of the developed method, it was applied to the analysis of CWA and related compounds in environmental water samples taken from the Baltic Sea. The 10 samples were taken from various locations in the Baltic Sea as part of the DAIMON project [39] and were stored in a freezer. They were then analyzed using the optimized SPE method. Using the optimized MOF-SPE-GC-MS/MS with obtained dMRM parameters, no CWA or related compounds were detected in the analyzed environmental samples. Speaking of the water samples was necessary to further assess the applicability of the optimized method. The recoveries of analytes were in the range presented in Table 3. These results show that the proposed method is suitable and repeatable.

### 2.6. Comparison with Other Methods

**Figure 13:**
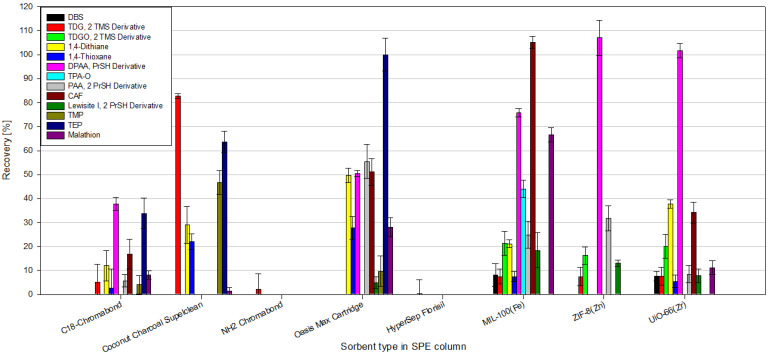
Comparison of recoveries of CWAs, their simulants, and degradation products from spiked water samples obtained using commercial SPE sorbents vs. MOFs.

According to our knowledge, no one has previously tested the feasibility of using MOFs as sorbents in the SPE technique for CWA determination. According to the information presented in the Introduction, most of the work on MOFs and CWAs is devoted to the absorption, degradation, and catalysis of the decomposition of CWAs or the creation of materials or filters that protect against these compounds. There are only a few articles in which the authors use MOFs for CWA analysis; the articles in question are summarized in Table 4. These articles focus more on the development of new prototype analytical techniques; they use only a few analytes focusing on 2-chloroethyl ethyl sulfide (CEES) or dimethyl methyl phosphonate (DMMP), often use spectrophotometric techniques to detect CWA, and the LODs achieved are much higher than those presented in this work. To the best of our knowledge, this article is the first to use the MOFs as sorbents in the classic SPE technique for the analysis of as many as 13 selected chemical warfare agents, their simulants, and degradation products in aqueous samples, in conjunction with the use of GC-MS/MS. For comparison (Table 4), several additional articles are presented in which the authors use MOFs as sorbents in the SPE technique for the determination of “similar” analytes in aqueous samples, e.g., PAHs, NSAIDs, and Sulphonamides, in a manner more similar to that presented in this paper. Moreover, a few additional articles where authors used different sorbents in the SPE technique for CWA determination in water samples are shown in Table 4. Moreover, during this study, the efficiency of extraction of CWAs, their simulants, and degradation products from water samples by the SPE technique using MOFs as sorbents and several commercially available sorbents were compared (Figure 13). The following commercial SPE columns were used for comparison: C18-Chromabond, Coconut Charcoal Supelclean, NH2-Chromabond, Oasis Max Cartridge, and HyperSep Florisil. Commercial SPE columns are available in many sizes, with different amounts of sorbent inside, which may affect the extraction results. To standardize the sample preparation conditions, identical, empty SPE columns were used to pour 100 mg of commercial and MOF sorbents, respectively. While determining the given compounds, the commercial columns of the NH2 type, Chromabond and HyperSep Florisil, performed the worst. The recoveries of the tested analytes were minimal; in most cases, they did not exceed 1%. No significant selectivity of the tested sorbents to the determined compounds was observed either. Oasis Max Cartridge shows the best results among commercial sorbents; relatively good recoveries were obtained for several tested compounds using this sorbent. Equally interesting are the results obtained with the use of MOFs. MIL-100(*Fe*) allows for the extraction of most of the tested compounds from the water sample. Excellent recoveries were obtained for CAP, DPAA, malathion, and TPAO. The use of ZIF-8(*Zn*) and UiO-66(*Zr*) seems less attractive than MIL-100(*Fe*); however, the use of the former allowed for excellent recoveries for DPAA.

Additionally, we compared the sorption capacities of the obtained MOFs with commercial materials. The results for most of the tested compounds are similar. However, some compounds such as DBS, Thioxane, or Dithiane were sorbed with approximately 10% higher efficiency on commercial MOFs.

### 2.7. Possible Mechanism for the Analytes Sorption on Selected MOFs

MOFs act as sorbents in several ways. The first is classical intermolecular interactions on the MOF surface. This is the same type of interaction as in classical sorbents, such as activated carbons. This effect can be more potent in MOFs through this material’s relatively larger specific surface area. The second way is the “sieving” of analytes on the pores of the MOF. MOFs are characterized by fixed, small pore sizes specific to the given structure. Smaller analytes will be able to “enter” inside the structure of the metal–organic framework, while larger ones will be sorbed only on their surface, as in the case of classical sorbents. In this study, the third way was the π-π interactions between the delocalized orbitals of MOFs’ organic ligands and determined analytes. For this study, MIL-100(*Fe*) proved to be the best sorbent, as, of the MOFs studied, it was the one that allowed for high recoveries for the most significant number of compounds tested. It is challenging to point out which of the three factors discussed had the most significant impact on the results obtained, as each certainly played a role here. The SciGress program was used to estimate the size of the analytes studied, and the estimated sizes of the compounds are shown in Table 5. Of particular notice here is the recovery of one of the compounds (malathion) obtained using MIL-100(*Fe*). The MIL showed an affinity for compounds containing aromatic rings in their structure (e.g., TPAO, CAP, DPAA), thanks to π-π interactions. However, malathion proved to be an exception to this rule. MIL-100(*Fe*) is characterized by two types of pores, the larger ones being of about 1.93 nm, while the estimated size of the malathion molecule is of 1.14 nm. The molecule of this analyte is noticeably more significant than the other compounds tested, and its size is best suited to the larger MIL-100(*Fe*) pore. The other two malathion-like compounds, TMP and TEP, were poorly recovered by this material. Although they are similar to malathion from a chemical structure perspective (similar chemical groups and their arrangement), they are also much less spatially extended than it. It was not the intermolecular interactions that were most important during the sorption of malathion in this case.

In our opinion, organic ligands exert the most significant influence on MIL-100(*Fe*) sorption properties, which differ from the other MOFs tested. It is thanks to these ligands that the interaction is possible. This assumption is confirmed by the fact that the compounds with the highest sorption efficiency contained aromatic rings. Additionally, it can be stated that MIL-100(*Fe*) should sorb well all compounds that have aromatic rings in their structure.

## 3. Experimental

### 3.1. Materials

#### 3.1.1. Apparatus and GC Conditions

Fourier Transform Infrared Spectrophotometer used: IRTracer-100, SHIMADZU (Kyoto, Japan), with QATR 10 attachment, Single Reflection ATR Accessory, and LabSolutions IR control and data processing software was used. Several scans per measurement-10, measurement resolution 4 cm^−1^, Happ-Genzel apodization (FT-IR/ATR).

Scanning electron microscope (SEM) images were obtained with a Quanta 3D scanning SEM-FEG. The XRD analysis was conducted using the Bruker D2 PHASER (Billerica, MA, USA) diffractometer with Cu Kα X rays operating at 30 kV and 10 mA, in the range of 3° < 2*θ* < 70° at room temperature.

Nitrogen adsorption isotherms were measured at −196 °C using the ASAP 2020 volumetric analyzer manufactured by Micromeritics Instrument Corp. (Norcross, GA, USA). Experimental errors associated with the measured adsorption data using commercial adsorption analyzers have been discussed elsewhere [51]. All samples were degassed at 150 °C for 12 h before adsorption measurements. The specific surface area was estimated using the Brunauer–Emmett–Teller (BET) method based on low-temperature nitrogen adsorption isotherms in a relative pressure (p/p_0_) range of 0.05–0.20 [52]. The total pore volume was calculated using the volume of the nitrogen adsorbed at a relative pressure of ≈0.99. The pore size distribution (PSD) functions were calculated from nitrogen adsorption isotherms by using the non-local density functional theory method (2D-NLDFT) for zeolites and siliceous materials with cylindrical pores because there is no NLDFT method dedicated especially to MOFs [53]. The calculations were performed using the numerical program SAIEUS developed by J. Jagiello (Micromeritics) [54].

Vacuum manifold used for SPE–J.T. Baker (Phillipsburg, New Jersey 8865. United States) SPE–12 G, Prod. No. 7520-94, connected to a vacuum pump—Laboport N 810.

Accessory equipment: HLP 5UV HYDROLAB (Straszyn, Poland) water demineralizer, Multi Reax Heidolph (Schwbach, Germany) shakers, M-Universal laboratory centrifuges, MPW Industries (Warsaw Poland), SONIC-10 POLSONIC (Warsaw Poland) ultrasonic cleaner, V02 vacuum dryer by Memmert (Schwbach, Germany).

Chromatographic analyses were conducted using a gas chromatograph (7890A GC System) coupled to a tandem mass spectrometer (7000 GC/MS Triple Quad), (GC-MS/MS), with an autosampler (GC Sampler 80) all from Agilent Technologies (Santa Clara, CA, USA). Rtx-5 column, Restek, 30 m length × 0.25 mm inner diameter × 0.25 µm film thickness. Helium was a carrier gas with a flow rate of 1 mL min^−1^. Data collection and processing were performed using MassHunter B.01.04 software (Agilent Technologies). GC analysis conditions: temperature of the inlet, 250 °C; the sample volume dispensed into the inlet, 1 µL in splitless mode. The analysis was carried out using a temperature program: the chromatographic column was kept for 1 min at 40 °C, then heated to 270 °C at a rate of 10 °C min^−1^ and held at the final temperature for 5 min. The actual analyses were carried out in dMRM (dynamic Multiple Reaction Monitoring) mode. The MS conditions were as follows: the temperatures of the transfer line, ion source, and quadruples were 280, 230, and 150 °C, respectively. The analysis was carried out using Electron Ionization (EI) with the standard 70 eV electron energy. N_2_ was used as a collision gas in MS/MS analysis, with a flow of 1.5 mL min^−1^ in the collision cell. The minimum dwell time was set to 50 ms. The transition from precursor ions to product ions of each analyte, collision cell energies, and other information necessary for operation in dMRM mode were determined using a stock solution of standards. The MRM transitions are summarized in Table S1.

#### 3.1.2. Reagents

N, N-Dimethylformamide (DMF, pure), Acetonitrile (ACN, p.a.), Acetone (p.a. basic 99.5%), dichloromethane (DCM, p.a. basic 99.8% (stabilized with amylene)), Methanol (MeOH, p.a.), HCl (36.5%, p.a.) from POCH, Gliwice, Poland. H_2_O deionized was obtained using water demineralizer HLP 5UV HYDROLAB, Bis(trimethylsilyl)trifluoroacetamide (BSTFA, SILYL-991, 95–100%) from Macherey-Nagel, Düren, Germany, 1-Propanethiol (PrSH, 98%) from Alfa Aesar, Haverhill, USA, Triphenylarsine oxide (TPA-O, pure, 97.0%), Thiodiglycol (TDG, pure) from Honeywell Fluka, Charlotte, NC, USA, malathion (99.3%) from Institute of Organic Industrial Chemistry, Warsaw, Poland, Thiodiglycol sulfoxide (TDGO), Diphenylarsinic acid (DPAA), Phenylarsonic acid (PAA), a-Chloroacetophenone (CAP), Chlorovinylarsine dichloride (Lewisite I) and Triethyl phosphate (TEP) were synthesized in small amounts in the laboratory, and their purity was checked using GC-MS, and it was ~95%, Commercial MOF (for reference), Basolite F300 (MIL-100(*Fe*)), Basolite Z1200 (ZIF-8(*Zn*)) from BASF, UiO-66(*Zr*) from ABCR, Karlsruhe, Germany, 1,4-Dithiane (97%), 1,4-Thioxane (97%), Trimesic acid (95%), Dimethyl sulfide (98%), Terephthalic acid (98%) from Sigma-Aldrich, Saint Louis, MO, USA, N-Butyl sulfide (DBS, pure, 99%), Trimethyl phosphate (TMP, 99%) from Acros Organics (part of Thermo Fisher Scientific), ZrCl_4_ (98%, anhydrous) from Thermo Fisher Scientific, Waltham, MA, USA, 2-methyl imidazole (98% for synthesis) from ROTH, Karlsruhe, Germany, Zinc Acetate dihydrate (97%), Zinc Oxide (pure, 99%), NaOH (p.a.), iron (II) chloride tetrahydrate (99%) from Warchem, Warsaw, Poland. Commercial sorbent for SPE columns: C18 Chromabond, Coconut Charcoal Supelclean, NH_2_ Chromabond, Oasis Max Cartridge, HyperSep Florisil. The analyzed CWAs, their simulants, and degradation products are presented in Table S2.

### 3.2. Methodology

#### 3.2.1. Preparation of MIL-100(Fe)

MIL-100(*Fe*) was obtained by a modified method presented in the literature [55]. Briefly, the process starts with the preparation of two different solutions. Solution 1 consisted of trimesic acid (1.676 g) and NaOH (0.912 g) dissolved in deionized water (22.808 g). Solution 2 consisted of FeCl_2_·4H_2_O (2.26 g) dissolved in deionized water (97.2 g). After completely dissolving all substrates in both solutions, solution 1 was added dropwise to solution 2 under stirring at room temperature. After combining both solutions, the molar ratios of substrates were 1.5 Fe: 1.0 trimesic acid: 3.0 NaOH: 880 H_2_O. The stirring continued at room temperature for 24 h. The solid was recovered and then washed three times with deionized water and one time with ethanol. The product was then vacuum dried at 120 °C overnight.

#### 3.2.2. Preparation of ZIF-8(Zn)

ZIF-8(*Zn*) was obtained by converting Hydroxy Double Salts (HDS) to MOF using metal oxide particles, according to the modified procedure presented in the literature [56]. Briefly, the synthesis of ZIF-8(*Zn*) from (Zn, Zn) Hydroxy Double Salts (HDS) was carried out at room temperature by mixing 5 mL of aqueous ZnO suspension (407 mg) (ZnO nano-suspension made using ultrasound) with 5 mL of aqueous Zn(CH_3_COO)_2_ (1098 mg) and 5 mL DMF for 24 h. Then, 3 mL of the (Zn, Zn) HDS suspension (made in the previous step) was added to 9 mL of 2-methylimidazole solution (493 mg). The mixture was stirred with a magnetic stirrer at room temperature for 10 min. The resulting product was vacuum filtered, washed several times with small portions of DMF, and dried for 2 h at 150 °C.

#### 3.2.3. Preparation of UiO-66(Zr)

UiO-66(*Zr*) was obtained by a modified method presented in the literature [57]. Briefly, a small spherical flask was filled with a 1: 1.14 molar ratio of ZrCl_4_ (0.54 mmol pre-dissolved in DMF-HCl (5:1 *v*:*v*) to terephthalic acid (pre-dissolved in 10 mL of DMF). The mixture was heated at 80 °C for 24 h. The product, a white precipitate, was vacuum-drained and washed several times with small portions of DMF and MeOH. The resulting UiO-66 was then vacuum-dried at 120 °C overnight.

#### 3.2.4. Preparation of Standard Solutions

Three sets of the standard solutions of the analytes under study in acetone were prepared. The first set, designated “Intact”, includes analytes that do not require derivatization for analysis using gas chromatography (these were the following compounds: DBS, 1,4-Dithiane, 1,4-Thioxane, TPA-O, CAP, TMP, TEP, malathion). The second set, labeled “BSTFA”, was a solution of analytes requiring derivatization with BSTFA (TDG, TDGO). The third is labeled “PrSH”—a solution of analytes requiring derivatization with PrSH (Lewisite I, PAA, DPAA). First, three stock solutions of analytes were prepared by dissolving each substance in acetone in a 25 mL volumetric flask to the concentration of 1 mg mL^−1^. As prepared, stock solutions were then diluted subsequently to 0.1 and 0.01 mg mL^−1^ in 10 mL volumetric flasks. Diluted stock solutions (at the concentration of 0.01 mg mL^−1^) were used to prepare standard solutions to determine the calibration curve during validation. Then, 0.01 mg mL^−1^ solutions were dissolved to, firstly, 1000 ng mL^−1^ in 50 mL volumetric flasks, and then 1000 ng mL^−1^ standard solutions were used to prepare 800, 500, 200, 100, 80, 50, 20, 10, and 1 ng mL^−1^ standard solutions in 10 mL volumetric flask. These standard solutions were used to determine the calibration curve during validation. All stock and standard solutions were kept at 4 °C in darkness.

#### 3.2.5. Sample Preparation

The method was optimized using spiked deionized water samples. After that, its feasibility was tested with the spiked environmental water samples from the Baltic Sea. Three parallel water samples were prepared by spiking 10 mL of deionized water with a dissolved stock solution of the test compounds dissolved in acetone, prepared in the previous step (100 µL of dissolved stock solution at 0.01 mg mL^−1^). Each water sample was spiked with only one standard solution, respectively, “Intact”, “BSTFA”, or “PrSH”. For example, to test the sorption character of MIL-100(*Fe*) against all analytes tested, it was necessary to prepare three separate samples of water spiked with the prepared solutions, “Intact”, “BSTFA”, and “PrSH”, and conduct three different analyses. The water samples were prepared in 15 mL Falcon-type plastic tubes. After spiking, the tubes were shaken manually for 15 s; then, a shaker was used for 10 min, and finally, they were subjected to an ultrasound for 5 min. Samples prepared this way were immediately subjected to further analysis using the SPE technique. An example of a sample preparation scheme is shown in Figure 1.

#### 3.2.6. SPE Procedure

Figure 1 shows the general procedure for sample preparation using the SPE technique wherein tested MOFs, MIL-100(*Fe*), ZIF-8(*Zn*), and UiO-66(*Zr*) in amounts of 150, 200, and 100 mg, respectively, were placed in empty plastic SPE columns between two porous disks. The SPE column thus prepared was then used to prepare the samples spiked in the previous step.

The columns were first conditioned by passing 2 mL of acetonitrile, and then 2 mL of H_2_O DI. During the conditioning step, special care was taken to leave a small amount of liquid over the surface of the sorbent each time. A 10 mL aqueous sample was then applied to the column. The stopcock regulation established a low sample flow velocity through the sorbent bed to prolong the analyte–sorbent bed interaction time. The next step was to dry the sorbent, i.e., let the column dry for 15 min in the flow of air sucked into the manifold through the column. The next step was to elute the analyte from the MOF sorbent bed using 2 mL acetonitrile in 2 × 1 mL portions. To analyze an aqueous sample contaminated with compounds not requiring derivatization, 1600 µL of the eluate was transferred to an autosampler vial and analyzed by GC-MS/MS. For compounds requiring derivatization, 1450 µL of eluate and 150 µL of BSTFA or PrSH as derivatizing reagents were placed in the autosampler vial. The vial containing the eluate with the appropriate derivatizing agent was heated at 60 °C for 1 h and then analyzed.

## 4. Conclusions

Three selected MOFs, MIL-100(*Fe*), ZIF-8(*Zn*), and UiO-66(*Zr*), were synthesized using facile methods and were then used as sorbents in the SPE technique to determine 13 selected CWAs, their simulants, and degradation products in water samples. A gas chromatograph with a tandem mass spectrometer was used during the experiments. The synthesized MOFs were characterized using FT-IR/ATR, SEM, XRD, and ASAP. The specific surface area for the ZIF-8(*Zn*), MIL-100(*Fe*), and UiO-66(*Zr*) was of 1479, 805, and 392 m^2^ g^−1^, respectively. The obtained sorbents, showing a high specific surface area and exhibiting a crystal structure, can be used as sorbents to determine CWA and related compounds in water samples. Additionally, we compared the sorption capacities of the obtained MOFs with commercial materials. The differences were not large, around 10%. As part of the research, MOF was compared with commercial SPE materials. From all of the tested commercial SPE phases, only “Oasis Max” shows relatively good sorption properties in relation to most of the tested compounds. However, Mil-100(*Fe*) will provide the highest recoveries of the three MOFs tested. The specific surface area for the ZIF-8(*Zn*), MIL-100(*Fe*), and UiO-66(*Zr*) was of 1479, 805, and 392 m^2^ g^−1^, respectively. The developed analytical method is suitable for determining the tested compounds in a wide range of concentrations with good precision. The best results were obtained for DPAA with ZIF-8(*Zn*) (LOD 0.62 ng mL^−1^, recovery 103.50%, precision 2.91%), CAP with MIL-100(*Fe*) (LOD 0.35 ng mL^−1^, recovery 100.23%, precision 4.74%), and malathion with MIL-100(*Fe*) (LOD 1.44 ng mL^−1^, recovery 66.84%, precision 3.00%). A wide spectrum of analytes was chosen for research in order to select a group of compounds that can be analyzed using the developed analytical procedure. Therefore, some of these analytes are efficiently sorbed onto the tested MOFs. However, some of the selected analytes show low recoveries, which was expected. MOFs probably catalyze the decomposition of some analytes, for example, as a result of hydrolysis, which was described in the literature [58]. In our opinion, MIL-100(*Fe*) turned out to be the best among the tested MOFs. It should be noted that the best results were obtained for compounds having at least one aromatic ring or a “spread” spatial structure. This work not only presented a simple method for the determination of selected CWAs, their simulants, and degradation products in water samples using MOFs as sorbents in the SPE technique, but also indicates further potential directions of using MOFs as sorbents overall.

## Figures and Tables

**Table 1:** The structural parameters of the synthesized MOFs. These parameters were determined based on low-temperature nitrogen adsorption isotherms. Additionally, selected parameters for the porosity of commercial MOFs are shown for comparison.

Synthesized								Commercial			
**MOF**	S_BET_ m^2^ g^−1^	V_t_ cm^3^ g^−1^	V_ultra_ cm^3^ g^−1^	V_micro_ cm^3^ g^−1^	V_meso_ cm^3^ g^−1^	Micro Porosity %	w nm	S_BET_ m^2^ g^−1^	Pore Opening nm	Pore Diameter nm	Ref.
ZIF-8(*Zn*)	1479	0.76	0.10	0.67	0.09	88	0.82	1626	0.34	1.16	[20]
MIL-100(*Fe*)	805	0.46	0.14	0.32	0.14	70	0.64 1.93	2155	0.5 0.9	2.5 2.9	
UiO-66(*Zr*)	392	0.33	0.15	0.19	0.14	58	0.55	1390	0.6	0.8 1.1	

S_BET_—BET specific surface area; V_t_—total (single-point) pore volume obtained from the amount adsorbed at p/p_0_ ≈ 0.99; V_ultra_—volume of ultramicropores (micropores smaller than 0.7 nm) obtained based on DFT PSD; V_micro_—volume of micropores (pores < 2 nm) obtained based on DFT PSD; V_meso_—the volume of mesopores (pores size between 2 and 50 nm) calculated by V_t_—V_micro_; w—average pore size; Microporosity—the percentage of the volume of micropores (V_micro_) to the total pore volume (V_t_); Pore opening—the pore window size; Pore diameter—the internal pore width.

**Table 2:** Validation parameters are specified for the GC-MS/MS method to determine chosen CWAs, their simulants, and degradation products.

Analyte	LOD ng mL^−1^	LOQ ng mL^−1^	Enrichment Factor (EF)	LOD with EF ng mL^−1^	LOQs with EF ng mL^−1^	Linear Range ng mL^−1^	R^2^	Precision (RSD, n = 7), 10 ng mL^−1^, (50 ng mL^−1^ for TPA-O) Intra-Day, %
DBS	2.11	6.33	5	0.42	1.27	1.27–1000	0.9981	7.13
TDG, 2 TMS Derivative	0.19	0.57	5	0.04	0.11	0.11–1000	0.9969	2.01
TDGO, 2 TMS Derivative	3.84	11.51	5	0.77	2.30	2.30–1000	0.9954	1.90
1,4-Dithiane	1.24	3.71	5	0.25	0.74	0.74–1000	0.9998	7.98
1,4-Thioxane	1.71	5.13	5	0.34	1.03	1.03–1000	0.9999	8.01
DPAA, PrSH Derivative	3.12	9.35	5	0.62	1.87	1.87–1000	0.9988	1.73
TPAO	37.69	113.08	5	7.54	22.62	22.62–1000	0.9997	8.45
PAA, 2 PrSH Derivative	1.32	3.97	5	0.26	0.79	0.79–1000	0.9981	1.18
CAP	1.77	5.30	5	0.35	1.06	1.06–1000	0.9974	8.32
Lewisite I, 2 PrSH Derivative	6.38	19.14	5	1.28	3.83	3.83–1000	0.9900	2.43
TMP	1.03	3.08	5	0.21	0.62	0.62–1000	0.9967	7.54
TEP	1.28	3.84	5	0.26	0.77	0.77–1000	0.9946	6.04
Malathion	7.22	21.66	5	1.44	4.33	4.33–1000	0.9996	5.98

**Table 3:** Determined recoveries using optimized SPE/GC-MS/MS method for spiked water samples analysis for CWA determination.

Analyte	MOF	Spiked ng mL^−1^	Recovery %	RSD (n = 5) %
DBS	MIL-100(*Fe*)	10	8.01	8.43
		20	8.23	7.99
TDG, 2 TMS Derivative	MIL-100(*Fe*)	10	7.35	10.14
		20	8.02	5.85
TDGO, 2 TMS Derivative	MIL-100(*Fe*)	10	20.58	5.11
		20	20.54	2.49
1,4-Dithiane	UiO-66(*Zr*)	10	40.12	6.90
		20	38.43	9.58
1,4-Thioxane	MIL-100(*Fe*)	10	7.14	7.54
		20	9.15	7.90
DPAA, PrSH Derivative	ZIF-8(*Zn*)	10	104.83	4.47
		20	102.17	1.35
TPAO	MIL-100(*Fe*)	50	42.77	4.30
		100	45.16	7.07
PAA, 2 PrSH Derivative	ZIF-8(*Zn*)	10	34.95	5.41
		20	30.15	10.15
CAP	MIL-100(*Fe*)	10	99.31	8.03
		20	101.15	1.45
Lewisite I, 2 PrSH Derivative	MIL-100(*Fe*)	10	19.61	7.67
		20	20.69	8.82
Malathion	MIL-100(*Fe*)	10	66.59	2.55
		20	67.08	3.44

**Table 4:** Comparison of the proposed method with different methods in the literature.

MOF/Sorbent	Analyte	Matrix	Method	LOD	Ref.
Eu or Gd@UiO-67(*Hf*) composite	MPA, DMMP, DIMP, DEMP, EMP, TEP, CEES, EtOH	Wastewater and Plants	Luminescent Sensing Experiment (Spectrophotometer)	0.4 ppm	[40]
The die-cut glass fiber filters treated with MOFs (HKUST-1, UiO-66, and 67)	GB, GD, GF, MPO	−	Paper spray mass spectrometry (PS-MS)	−	[41]
UiO-67, 67-NH_2_ and 67-CH_3_	DMMP	−	Computational and practical study of adsorption capabilities	−	[42]
Zr-BTC	CEES	−	UV-Vis spectrometer	48 ppb	[43]
UiO-66	DMMP	Gas samples	Portable gas sensing, MEMS-MOF, FBAR sensor	2.64 ppm	[44]
UiO-66, and ZrQ@UiO-66	DMMP	Ethanol	Fluorescence quenching	8.3 nM	[45]
MOF-5	PAHs	Environmental water	HPLC-FLD	0.4–40 ng/L	[46]
MIL-101(***Cr***)@Graphene hybrid aerogel	NSAIDs	Deionized water, Tap water	HPLC-UV-Vis	0.01–0.10 ng/mL	[29]
MIL-101(*Cr*) and (*Fe*)	4 sulphonamides	Environmental water	UPLC-MS/MS	0.03–0.08 µg/L	[47]
Polymeric (MAA + EGDMA)	Sulfur and Nitrogen mustards	Non-polar organic mediums	GC-MS	0.075–0.150 µg/mL	[48]
Polymeric, Poly(MAA-co-EGDMA)	Nerve agents and organophosphorus esters	Non-polar organic matrices (n-hexane)	GC-MS	0.015–0.075 µg/mL	[49]
Carbon aerogel (CA)	10 degradation products of HD	Environmental water samples	HPLC-DAD and CE-DAD	0.17–0.50 µM	[50]
MIL-100(*Fe*), ZIF-8(*Zn*), UiO-66(*Zr*)	13 CWAs, their degradation products, or simulants	Water	GC-MS/MS	0.04–7.54 ng/mL	This work-study

**Table 5:** Estimated analyte sizes using SciGress 3.6.0 software.

No.	Analyte	Estimated Particle Size (Length) nm
1	DBS	1.22
2	TDG	0.92
3	TDGO	0.91
4	1,4-Dithiane	0.53
5	1,4-Thioxane	0.50
6	DPAA (Clark I)	0.93
7	TPA-O	0.98
8	PAA	0.72
9	CAP	0.79
10	Lewisite I	0.56
11	TMP	0.61
12	TEP	0.88
13	Malathion	1.14

## Data Availability

Dataset available on request from the authors.

## Supplementary Materials

The following supporting information can be downloaded at: https://www.mdpi.com/article/10.3390/molecules29143259/s1.
