# Supplementary material for: Novel Application of Metal–Organic Frameworks as Efficient Sorbents for Solid-Phase Extraction of Chemical Warfare Agents and Related Compounds in Water Samples
Source: Molecules. 2024 Jul 10;29(14):3259. doi: 10.3390/molecules29143259 (PMC11279877; doi:10.3390/molecules29143259)
Supplement: Supplementary file 1 [file molecules-29-03259-s001.zip › molecules-3067082-supplementary.pdf]

Table S1. MRM transitions of analyzed compounds.

Analyte	Precursor Ion	Product Ion	Average Dwell time ms	Collision Cell Energy eV
DBS	146	61	68.2	20
	146	56		10
	61	46		20
	54	41		10
TDG, 2-TMS Derivative	116	73	132.5	11
	116	101		8
	147	73		11
TDGO, 2-TMS Derivative	117	73	132.5	12
	117	45		8
	73	45		12,5
1,4-Dithiane	120	61	94.4	15
	120	105		8
	92	46		11
1,4-Thioxane	104	61	79.4	9
	104	74		7
	104	46		11
DPAA, PrSH Derivative	261	183	132.3	14
	261	107		13
	104	46		17
TPA-O	306	152	99.0	10
	227	152		25
	152	77		25
	152	151		15
PAA, 2 PrSH Derivative	227	107	132.4	13
	302	259		5
	227	185		9
CAF	105	77	132.5	23
	154	105		37
	105	51		2
Lewisite I, 2 PrSH Derivative	286	176	132.5	8
	286	165		12
	286	43		20
TMP	140	79	56.3	20
	110	79		10
	95	65		5
	79	47		10
TEP	182	99	74.1	15
	155	99		10
	127	81		25
	99	81		15
Malathion	173	99	99.1	15
	158	125		5
	127	99		5
	99	43		15

Table S2. Determined compounds—CWA, their simulants, and degradation products.

No.	Analyte	Structure	Description	CAS
1.	Dibutyl sulfide (DBS)	S	Simulant of Sulphur mustard	544-40-1
2.	Thiodiglycol (TDG)	HO S OH	Hydrolysis product of Sulphur mustard	111-48-8
2.1.	Bis(trimethylsilyl)thiodiglycol, (TDG, 2 TMS Derivative)	Si O S O S	BSTFA derivative of 2.	20486-03-7
3.	Thiodiglycol sulfoxide (TDGO)	O S HO OH	The oxidation product of Sulphur mustard	3085-45-8
3.1.	Bis(trimethylsilyl)thiodiglycol oxide, (TDGO, 2 TMS Derivative)	O Si O S O Si	BSTFA derivative of 3.	97916-03-5
4.	1,4-Dithiane	S S	Degradation product of Sulphur mustard	505-29-3
5.	1,4-Thioxane	S O	Degradation product of Sulphur mustard	15980-15-1
6.	Diphenylarsinic acid (DPAA)	O As OH	The oxidation product of Clark I and Clark II and all of their degradation products (either natural or with H_2O_2)	4656-80-8
6.1.	Diphenylpropylthioarsine, (DPAA, PrSH Derivative)	S As	PrSH derivative of 6.	17544-92-2
7.	Triphenylarsine oxide (TPA-O)	O As	The oxidation product of Triphenylarsine	1153-05-5
8.	Phenylarsonic acid (PAA)	OH O As OH	The oxidation product of Phenyldichloroarsine (PDCA) and all of its degradation products (either natural or with H_2O_2)	98-05-5
8.1.	Dipropyl phenylarsonodithioite, (PAA, 2 PrSH Derivative)	S As S	PrSH derivative of 8.	1776-69-8
9.	a-Chloroacetophenone (CAF)	O Cl	CWA agent	532-27-4
10.	Chlorovinylarsine dichloride (Lewisite I)	Cl As Cl Cl	CWA agent	541-25-3

No.	Analyte	Structure	Description	CAS
10.1.	Dipropyl (2-chlorovinyl)arsonodithiolite, (Lewisite I, 2 PrSH Derivative)	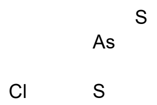	PrSH derivative of 10.	677354-97-1
11.	Trimethyl phosphate (TMP)	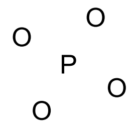	Degradation product of Organophosphate CWA	512-56-1
12.	Triethyl phosphate (TEP)	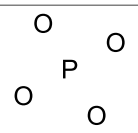	Degradation product of Organophosphate CWA	78-40-0
13.	2-(Dimethoxyphosphinothioylthio) butanedioic acid diethyl ester (Malathion)	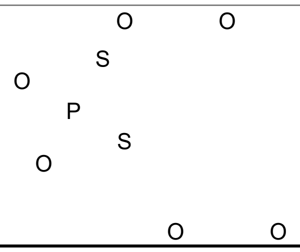	Simulant of Organophosphate CWA	121-75-5

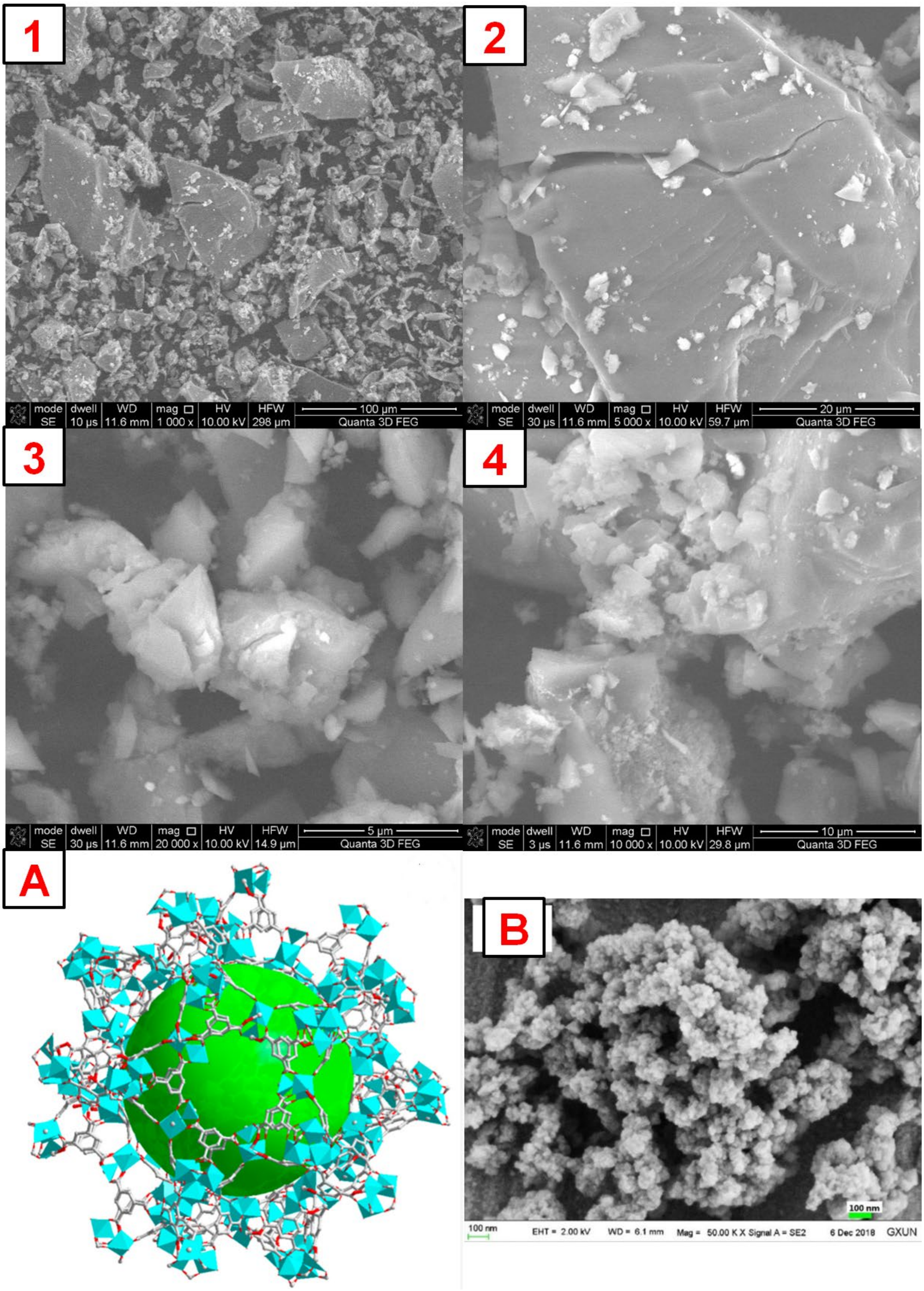

Figure S1. 1, 2, 3 and 4—SEM photos of the MIL-100(Fe) obtained. For comparison: A—a schematic representation of the MIL-100(Fe) structure, the green sphere inside the structure represents the space inside the pore (44), and B—an SEM photo found in the literature [41].

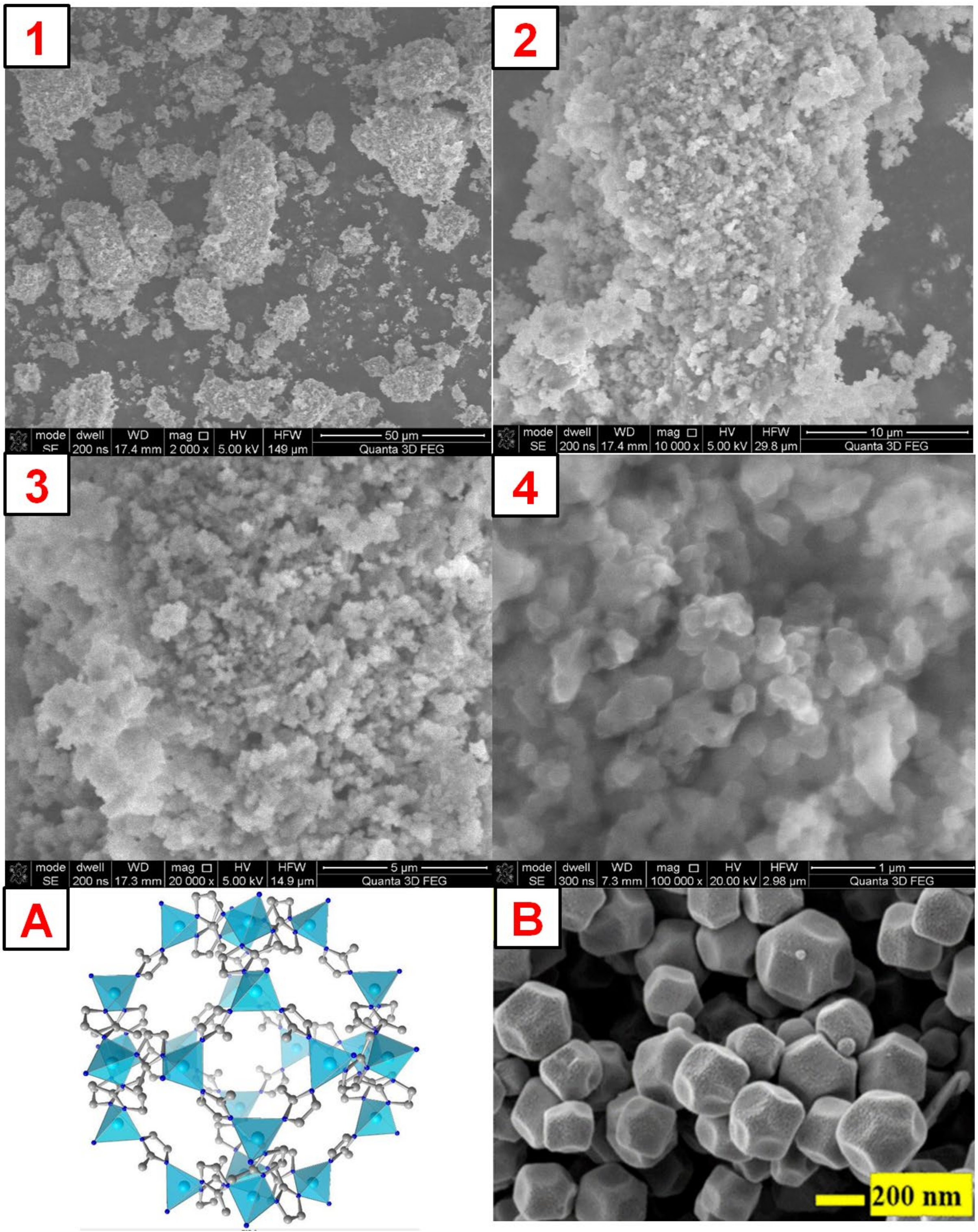

Figure S1. 1, 2, 3 and 4—SEM photos of the obtained ZIF-8(Zn). For comparison: A—a schematic representation of the ZIF-8(Zn) structure [45], and B—a SEM photo found in the literature [42].

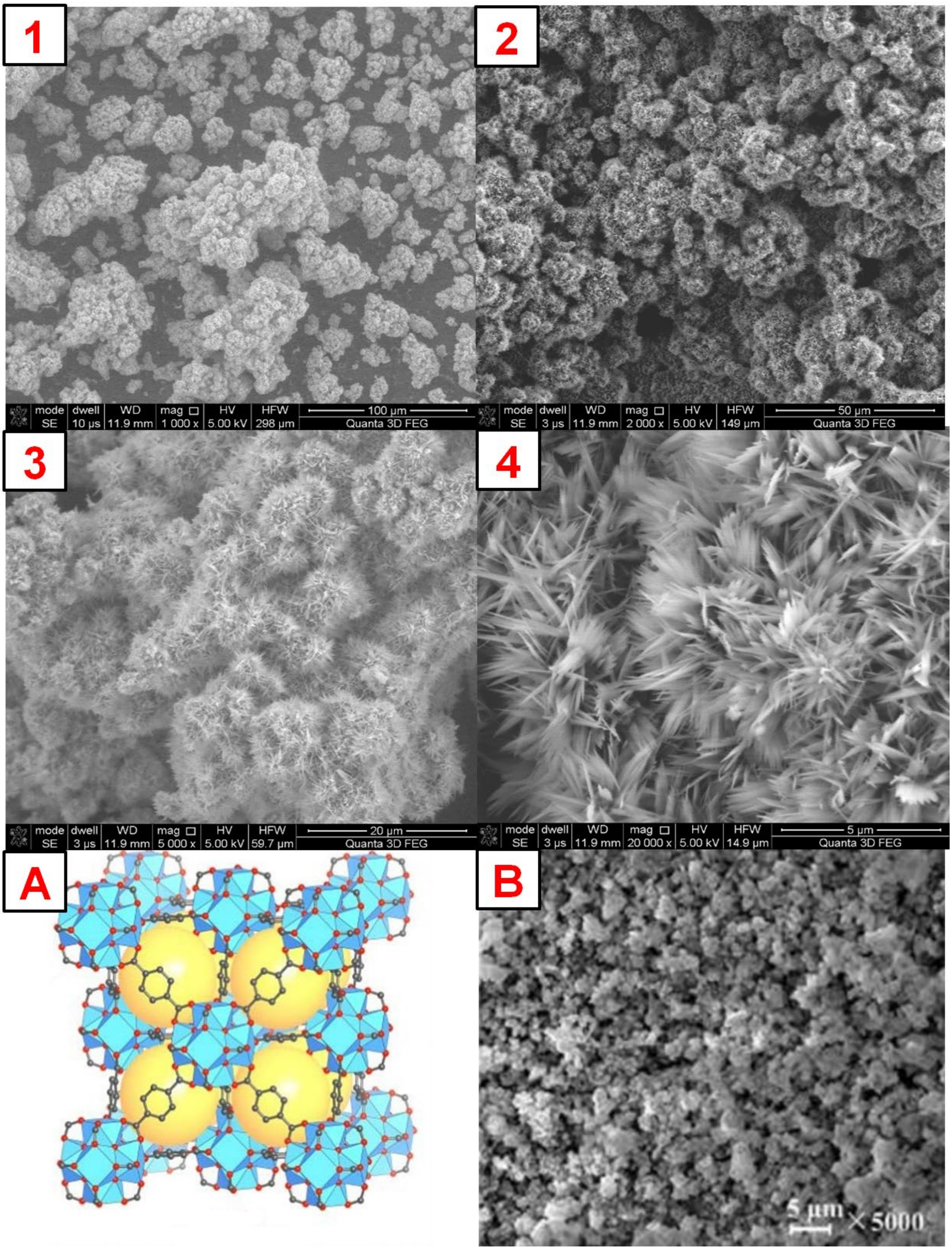

Figure S2. 1, 2, 3 and 4—SEM photos of the obtained UiO-66(Zr). For comparison: A—a schematic representation of the UiO-66(Zr) structure, the yellow sphere inside the structure represents the space inside the pore [20], and B—a SEM photo found in the literature [43].
